# Hyperoside: A Review of Its Structure, Synthesis, Pharmacology, Pharmacokinetics and Toxicity

**DOI:** 10.3390/molecules27093009

**Published:** 2022-05-07

**Authors:** Sijin Xu, Shuaipeng Chen, Wenxin Xia, Hong Sui, Xueyan Fu

**Affiliations:** 1 School of Pharmacy, Ningxia Medical University, Yinchuan 750004, China; 18931127856@163.com (S.X.); csp17695170414@163.com (S.C.); nxxwx183@163.com (W.X.); 2 Ningxia Collaborative Innovation Center of Regional Characteristic Traditional Chinese Medicine, Yinchuan 750004, China; 3 Key Laboratory of Ningxia Ethnomedicine Modernization, Ministry of Education, Ningxia Medical University, Yinchuan 750004, China; 4 Ningxia Regional Key Laboratory of Integrated Traditional Chinese and Western Medicine for Prevention and Treatment of Regional High Incidence Disease, Yinchuan 750004, China

**Keywords:** hyperoside, structure, pharmacology, pharmacokinetics, toxicity, review

## Abstract

Hyperoside is an active ingredient in plants, such as *Hypericum monogynum* in Hypericaceae, *Crataegus pinnatifida* in Rosaceae and *Polygonum aviculare* in Polygonaceae. Its pharmacologic effects include preventing cancer and protecting the brain, neurons, heart, kidneys, lung, blood vessels, bones, joints and liver, among others. Pharmacokinetic analysis of hyperoside has revealed that it mainly accumulates in the kidney. However, long-term application of high-dose hyperoside should be avoided in clinical practice because of its renal toxicity. This review summarises the structure, synthesis, pharmacology, pharmacokinetics and toxicity of hyperoside.

## 1. Introduction

**Figure 1:**
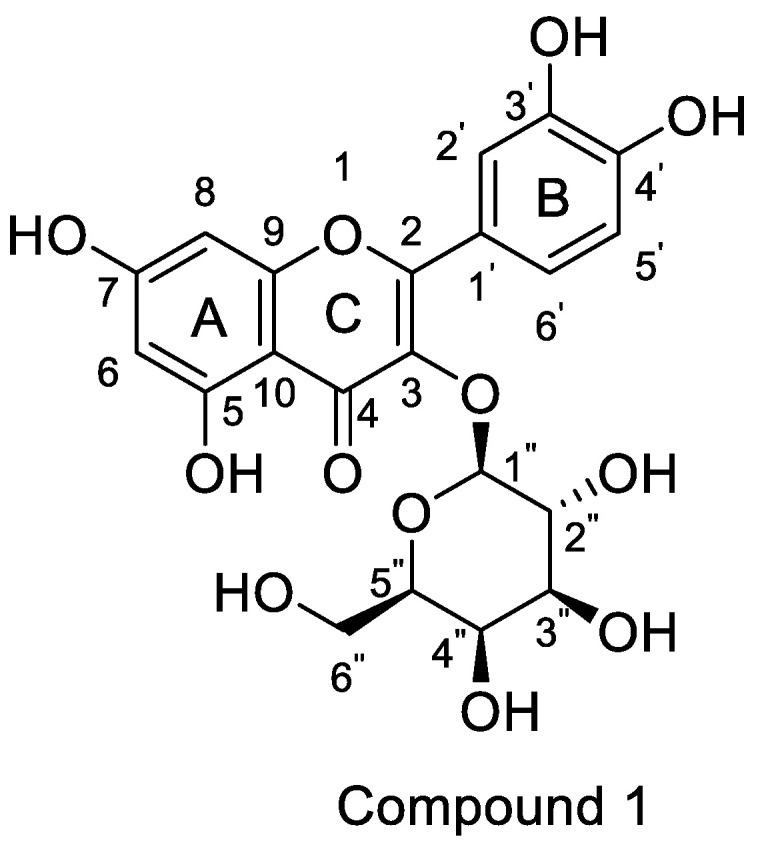
Structure of hyperoside.

Hyperoside, which has the structure shown in Figure 1, is an active ingredient in plants such as *Hypericum monogynum* in Hypericaceae, *Crataegus pinnatifida* in Rosaceae and *Polygonum aviculare* in Polygonaceae [1,2,3]. These plants are widely distributed worldwide, especially in Southeast Asian countries, such as China, Japan and South Korea. They exhibit various pharmacological effects, such as protecting the blood vessels, regulating the digestive system and protecting against oxidation, aging and cancer [4,5,6].

Hyperoside was first extracted and isolated from *Hypericum perforatum* L. in 1937 [7]. Considering the low content and difficult extraction of this compound, scholars usually employ chemical and biosynthetic methods to obtain hyperoside. Guna et al. used resting cell fermentation and recycling to increase hyperoside production in 2020, and for the first time, obtained a maximum yield of 18,000 mg/L [8]. Hyperoside, also known as quercetin 3-O-beta-D-galactopyranoside, is a yellow solid, and its aglycon is quercetin [9,10]. Its antioxidant activity may be related to the hydroxyl groups on the A and B rings and the glycosides linked to the C ring [11], whereas its analgesic effect may be related to 3-galactopyranoside [12]. It also has a high affinity for soy protein [13]. Considering these properties, Wang et al. fabricated and characterised zein–tea polyphenol–pectin ternary complex nanoparticles and zein–pectin composite nanoparticles as effective delivery systems for hyperoside [14,15]. Such systems undoubtedly greatly improve the bioavailability of hyperoside. Hyperoside exerts its anti-cancer and brain-, nerve- and kidney-protective functions through the phosphatidylinositol-3-kinase (PI3K)/protein kinase B (AKT) and nuclear factor E2-related factor 2 (Nrf2)/haem oxygenase-1 (HO-1) pathways. However, hyperoside easily accumulates in the kidney. Toxicity tests have shown that long-term use of hyperoside has nephrotoxic effects, but researchers have also found that this damage is reversible. So far, the toxic mechanism of hyperoside remains to be elucidated. This review summarises the structure, synthesis, pharmacology, pharmacokinetics and toxicity of hyperoside. This review may serve as a basis for developing hyperoside and expanding its application.

## 2. Structure and Synthesis

The aglycone of hyperoside is quercetin. Quercetin has many derivatives, such as quercetin-3′-O-acetic acid methyl ester, quercetin-3′-O-acetic acid, quercetin- 5-O-formate methyl ester and quercetin-5-O-formate [16]. Hyperoside (IUPAC name: 2-(3,4-dihydroxyphenyl)-5,7-dihydroxy-3-[(2S,3R,4S,5R,6R)-3,4,5-trihydroxy-6-(hydroxymethyl)oxan-2-yl]oxychromen-4-one) is a derivative of quercetin. Hyperoside also has derivatives, such as 2-(2,2-diphenyl-benzo [1,3] dioxincyclo-5-yl)-3-o-β-d-tetraacetyl pyrangalactose-5,7-dihydroxy-benzo pyran-4-one. Molecular docking of HCOV-229E showed that it has a good binding effect on the 3CL protease of HCOV-229E anti-prototype coronavirus [17].

### 2.1. Chemical Synthesis

**Scheme 1:**
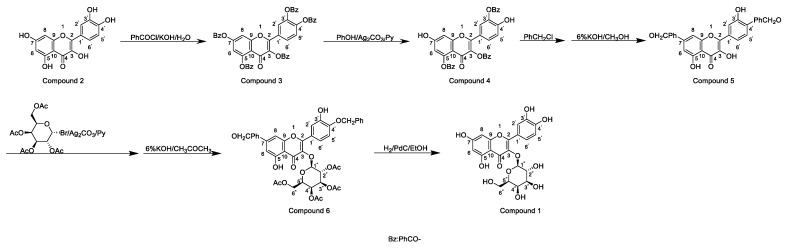
Hyperoside synthesis using a chemical method from the study by Horhammer et al. [18].

**Scheme 2:**
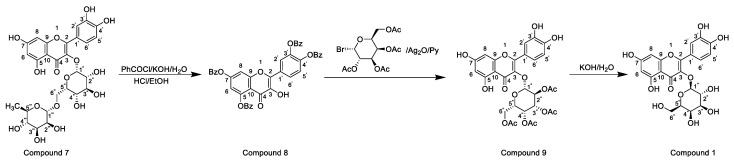
Hyperoside synthesis using a chemical method from the study by Jiang et al. [19].

**Scheme 3:**
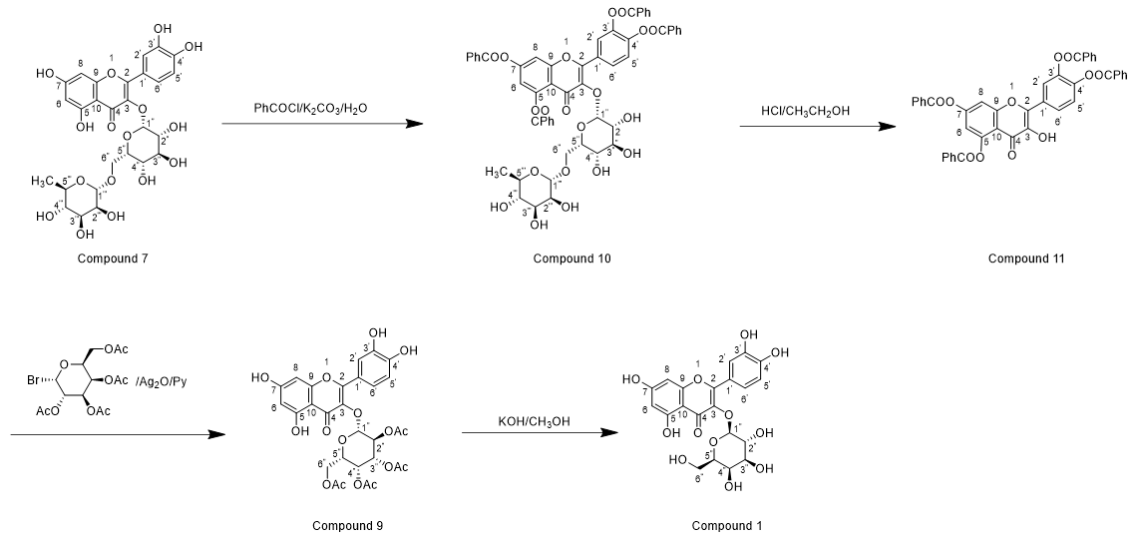
Hyperoside synthesis using a chemical method from the study by Zhou [12].

Horhammer et al. were the first to synthesise hyperoside. They used quercetin as a raw material for total synthesis and obtained a total yield of only 2.6%. The main synthetic challenge within the Horhammer synthesis of hyperoside was the selective glycosylation of the C3 hydroxyl group of quercetin. The series of highly controlled regioselective protection and deprotection operations resulted in the formation of compound 5. The Koening–Knorr reaction with this compound and acetylated bromoglucose proceeded regioselectivity at the hydroxyl in position C3. Further hydrogenolysis with hydrogen, using palladium on carbon as a catalyst, resulted in the formation of hyperoside [18]. This synthetic route is shown in Scheme 1. Following simple steps, Jiang et al. semi-synthesised hyperoside using rutin as the raw material under mild reaction conditions and obtained a total yield of 6.8% [19]. There are two things to note about this synthesis scheme. First, the team used compound 7 to react with benzoyl chloride and hydrolyse rutinose at the C3 position in the HCl/EtOH system to obtain compound 8. Second, the team also used the same reaction method as the previous team (the Koening–Knorr reaction) to select and further glycosylate the hydroxyl group at the C3, and the 5,7,3′ and 4′ substituents were converted to hydroxyl groups. Hyperoside was also obtained by hydrolysis of compound 9 [19] and the synthetic route is shown in Scheme 2. In 2002, Zhou integrated and improved the previous two schemes. Since the o-glycoside bond was usually a hemiacetal structure that can be easily hydrolysed, Zhou dissolved compound 10 in hot ethanol solution and hydrolysed it with strong HCl to obtain compound 11. He also used a Koening–Knorr reaction to remove the protective group in a KOH/anhydrous methanol system to obtain the target product. The hydrolysis conditions of benzoylated rutin were improved, and the yield of hyperoside was increased from 6.8% to 11% [12]. This synthetic route is shown in Scheme 3.

### 2.2. Biosynthesis

Guna et al. constructed a uridine 5′-diphosphate-galactose synthesis pathway in 2020 and synthesised hyperoside by using quercetin as a raw material. In this method, quercetin is added to uractose diphosphate galactose and *Escherichia coli* containing the *flavonol 3-O-galactosyltransferase* gene to produce uractose diphosphate and hyperoside. The supply of uractose diphosphate galactose to the recombinant strain must be improved to increase hyperoside yield. The optimal conversion temperature is 30 °C, but this parameter is affected by oxygen content. Eight layers of gauze can be used to increase oxygen supply and facilitate its synthesis. Moreover, resting cell fermentation and recycling can increase the yield of hyperoside. The maximum yield of hyperoside can reach 18,000 mg/L, which is 393% of batch fermentation, achieving production at a scale of 10 g/L for the first time [8]. This method has high output, but its expansion to large-scale production faces certain difficulties because of the complicated procedures and expensive equipment.

## 3. Pharmacology

Hyperoside exerts a wide range of pharmacological effects (Table 1, Table 2, Table 3, Table 4, Table 5, Table 6, Table 7, Table 8, Table 9, Table 10 and Table 11), such as preventing cancer and protecting the brain, neurons, heart and kidney, and by regulating various signalling pathways, metabolic processes, cytokines and kinases.

### 3.1. Anti-Cancer Activity

**Figure 2:**
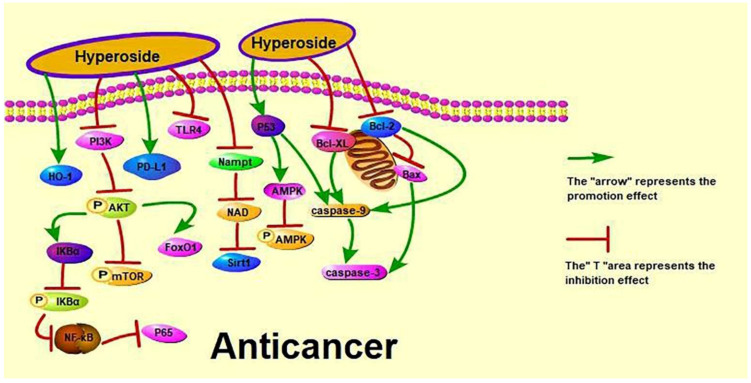
Anti-cancer effect of hyperoside.

The incidence and mortality of cancer have increased with industrialisation [111]. Lung cancer has received increasing attention because of its high incidence rate. Previous studies reported that hyperoside has anti-lung cancer effects and its mechanism is shown in Figure 2.

### 3.2. Effect on Lung Cancer

According to the World Health Organization (https://www.who.int/cancer accessed on 10 January 2022), lung cancer deaths have risen significantly in upper-middle-income countries to more than twice that of the three other income groups combined. Clinically, chemotherapy can slightly prolong the survival of patients with advanced cancer but at the cost of significant adverse reactions [112]. Recent studies have shown that hyperoside can induce the apoptosis and G1/S phase arrest and inhibit the proliferation of A549 cells by down-regulating the expression of B cell lymphoma-2 (Bcl-2) and B cell lymphoma-extra large (Bcl-xL) and up-regulating the expression of cysteinyl aspartate specific proteinase 3 (caspase-3) [27]. Dong et al. discussed the anti-cancer effects of hyperoside on non-small cell lung cancer (NSCLC) from different aspects. Dong et al. showed that hyperoside inhibits the expression of PD-L1 in NSCLC cells and at the cell membrane surface at the transcriptional level by reducing the protein expression of transcription factor cellular-myelocytomatosis viral oncogene (c-Myc) [24]. Chen et al. investigated the effect of hyperoside on hypoxia-induced NSCLC A549 cells and found that hyperoside increases the phosphorylation and HO-1 expression of A549 AMPK [25]. Furthermore, hyperoside can inhibit the proliferation and induce the apoptosis of T790M-positive NSCLC cells by up-regulating forkhead box protein O1 by colon cancer associated transcript 1. It can also inhibit the proliferation and induce the apoptosis of H1975 cells in a dose-dependent manner [26]. However, the effect of hyperoside on NSCLC remains unclear to date, as is its effect on small cell lung cancer.

### 3.3. Effect on Cervical Cancer

Cervical cancer is the most common gynaecological cancer in Brazil, second only to breast cancer in women [113]. In Japan, two cases of lung cancer in children (23-month-old and 6-year-old boys) were found to be caused by the mother-to-child transmission of a cervical tumour [114]. Bian et al. and Wang et al. have performed anti-cervical cancer experiments on HeLa cells. Bian et al. explored the effects of hyperoside on nicotinamide phosphoribosyltransferase (Nampt)/nicotinamide adenine dinucleotide (NAD)/silent information regulator 1 (Sirt1) expression during cell proliferation and migration and found that hyperoside treatment significantly decreased the mRNA expression levels for Nampt, NAD and Sirt1 [21]. Wang et al. studied the effects of hyperoside on the apoptosis and antioxidant capacity of HeLa cells by treating and culturing HeLa cells with hyperoside at 0, 25, 50, 100 and 200 μmol/L in vitro for 12, 24 and 48 h, and found that hyperoside decreased the cell survival rate in a dose- and time-dependent manner according to 3-(4,5)-dimethylthiahiazo(-z-yl)-3,5-di-phenytetrazoliumromide (MTT) assay, cell morphological observation, cell apoptosis detection and other methods. Their results also showed that hyperoside increases superoxide dismutase (SOD), catalase (CAT) and glutathione (GSH) activities; significantly decreases the expression of vascular endothelial growth factor (VEGF) and Bcl-2; and significantly increases the expression of malondialdehyde (MDA), Bax (Bcl2-associated X) and tumour suppressor gene p53 [20]. Guo et al. explained the effect of hyperoside on cervical cancer cells through a protein–protein interaction (PPI) network, PPI module analysis, transcription factor (TF)-target network construction and survival analysis, RT-QPCR and Western blot to detect key genes. They found that hyperoside down-regulates c-Myc gene expression and inhibits HeLa and C-33A cell proliferation [22].

### 3.4. Effect on Liver Cancer

Liver cancer is a high-risk cancer with a high fatality rate. Patients often neglect treatment or misdiagnose this disease at the initial stage because its symptoms are similar to those of other liver diseases. Moreover, liver cancer has become a severe disease affecting the average life expectancy in China [115]. Jiang et al. and Wei et al. studied the protective effect of hyperoside on human liver cancer cells through different pathways. Jiang et al. studied the effects of hyperoside on the apoptosis of human hepatoma HepG2 cells via the P53/caspase pathway, and found that this compound significantly increases (*p* < 0.05) the expression levels of P53, caspase-9 and caspase-3 proteins in HepG2 cells [23]. Wei et al. studied the effect of hyperoside on the PI3K/AKT pathway in human hepatocellular carcinoma cells. They found that hyperoside down-regulates the expression of bone morphogenetic protein 7 (BMP-7), arrests the cell cycle growth of HepG2 cells in the G1 phase, inhibits the phosphorylation of AKT and significantly down-regulates the expression of PI3K [6]. In addition, Han Jingxia and Hu et al. performed protein–protein interaction experiments, such as fast protein liquid chromatography, co-immunoprecipitation and metabolomics, to illustrate the anti-liver cancer effect of hyperoside. Han Jingxia found that hyperoside inhibits the interaction between YY1 and P65 and P300, reduces the activity of quaking (QKI) promoter and down-regulates the expression of has_circ_0004631 [116]. Hu et al. found that hyperoside at 60 mg/kg can prevent liver damage caused by acetaminophen-induced oxidative stress and regulate glutathione-related metabolites and enzymes by inhibiting cytochrome P450 2E1 [117]. In conclusion, hyperoside exerts its anti-liver cancer effect by inhibiting the proliferation of liver cancer cells, arresting their cell cycle and effectively inhibiting the activity of the YY1 complex, the expression of QKI and the invasion and metastasis of liver cancer cells.

### 3.5. Effect on Breast Cancer

In recent years, the incidence of breast cancer in China has gradually increased, and breast cancer has become the most common malignant tumour in women [118]. Qiu et al. and Sun et al. studied the effect of hyperoside on breast cancer. Qiu et al. investigated the effect of hyperoside on the apoptosis of breast cancer cells via the reactive oxygen species (ROS)-mediated nuclear factor kappa-B (NF-κB) pathway and found that hyperoside inhibits the survival and migration and promotes the apoptosis of MCF-7 and 4T1 cells [28]. Sun et al. showed that hyperoside inhibits cell viability and increases apoptosis and caspase-3 activity in toll-like receptor 4 (TLR4)-positive breast cancer MDA-MB-231 cells, enhancing the sensitivity of such cells to paclitaxel [29].

### 3.6. Effect on Stomach Cancer

The incidence of gastric cancer is decreasing, but its morbidity and mortality remain high. Liu Haiwen and Wang et al. studied the effect of hyperoside on the proliferation and apoptosis of human gastric cancer MKN-45 cells. Liu Haiwen used hyperoside doses of 25, 50 and 100 μg/mL, and Wang et al. used hyperoside doses of 50, 75 and 100 μg/mL [30,31]. Both studies found that hyperoside increases the apoptosis rate and the G0/G1 phase ratio; up-regulates caspase-3, Bax, nuclear factor B inhibitor (IκBα) expression; decreases M/G2/M phase cell ratio; and down-regulates NF-κB P65 and Bcl-2 protein expression [30,31]. However, in terms of toxicity, a lower dose of hyperoside is safe since it reduces kidney accumulation in vivo. Therefore, given its effectiveness against gastric cancer cells, 25 μg/mL hyperoside should be given priority in the treatment of this disease.

### 3.7. Effect on Other Cancers

Ovarian cancer has a very high mortality rate among gynaecological cancers and is a major threat to women’s health. Xu et al. showed that hyperoside can up-regulate cleaved-caspase-3 and caspase-9, down-regulate Bcl-2, reduce the protein levels of p65 and p-IκB-α and suppress the migration and invasive abilities of SKOV3 cells. It can also inhibit the activation of the NF-κB signalling pathway and resist ovarian cancer. Pancreatic cancer is a tumour of the digestive system [32]. Xue et al. found that hyperoside exerts a high killing activity on PANCI cells by inducing a high level of perforin in NK cells [33]. Skin cancer is primarily diagnosed visually, but dermatoscopic analysis, biopsy and histopathology are needed for confirmation [119]. Kong et al. studied the effect of hyperoside on skin tumours induced by 7, 12 dimethylbenz(a)anthracene (DMBA)/12-Otetradecanoylphorbol-13-acetate (TPA) and found that hyperoside can reduce the phosphorylation of PI3K, AKT, mammalian target of rapamycin (mTOR) and AMPK [34].

### 3.8. Brain Protection Activity

**Figure 3:**
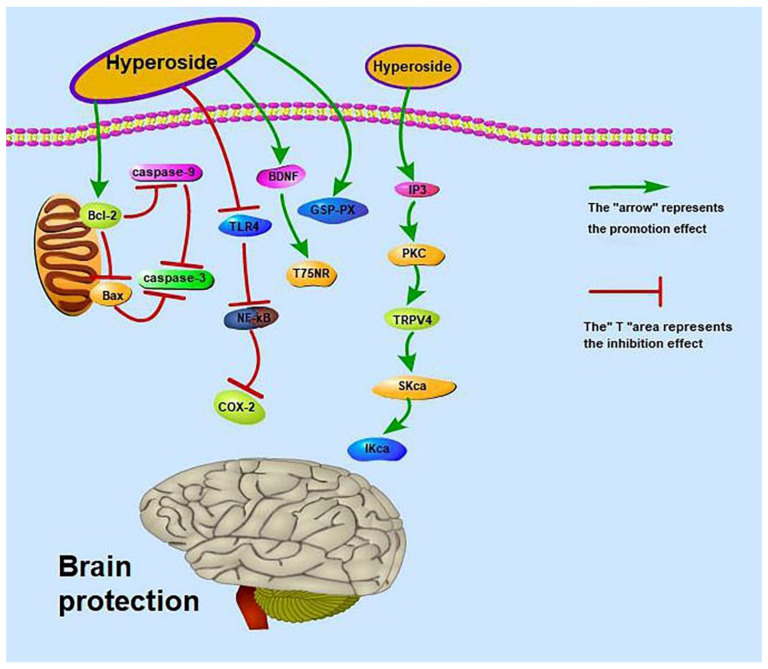
Brain-protective effect of hyperoside.

Brain-related diseases include cerebral ischaemia, stroke and so on. Hyperoside can regulate the expression of cerebral blood vessel transient receptor potential vanilloid 4 (TRPV4) by initiating the inositol trisphosphate (IP3)/protein kinase 1 (PKC) signalling pathway and activating intermediate conductance Kca (IKca) and small conductance (SKca) channels, thereby promoting Vascular endothelium-dependent hyperpolarization factor (EDHF) to generate vasodilation responses to improve ischaemic brain injury [70]. Hyperoside can also protect the brain and improve ischaemia–reperfusion. Ischemia–reperfusion is related to the TLR4/cyclooxygenase-2 signalling pathway and to brain-derived neuro-trophic factor (BDNF), p75 neurotrophin receptor (p75NRT), Bcl-2 mRNA, p-PI3K, p-AKT, Bax and caspase-3 [69,71,72]. Moreover, hyperoside can improve nickel-induced brain injuries [73]. The mechanisms by which hyperoside exerts brain protection are shown in Figure 3.

Some animal experimental data are also worthy of our attention. A previous study found that the intragastric administration of hyperoside at 50 mg/kg/day to Sprague–Dawley rats significantly decreases (*p* < 0.01) the cerebral infarct volume ratio; significantly increases the activities of total antioxidant capacity (T-AOC) (*p* < 0.01), superoxide dismutase (SOD) (*p* < 0.01) and glutathione peroxidase (GSH-Px) (*p* < 0.05); and significantly decreases the content of malondialdehyde (MDA) (*p* < 0.01) [72]. Intragastric administration of hyperoside at 25 and 12.5 mg/kg to rats also increases cerebral blood flow in the cerebral cortex [120]. These results suggest that hyperoside exerts a protective effect on cerebral infarction in rats. Intraperitoneal injection of 50 and 100 mg/kg hyperoside increases the activity of lactate dehydrogenase in the brain tissues of mice to 147.7 ± 20.4 (*p* < 0.01) and 163.3 ± 34.2 (*p* < 0.01), respectively, and improves learning and memory disorders in the platform test [121]. However, the drug doses used in this study are too large and may affect the metabolism and kidney function of mice.

### 3.9. Neuroprotective Effect

**Figure 4:**
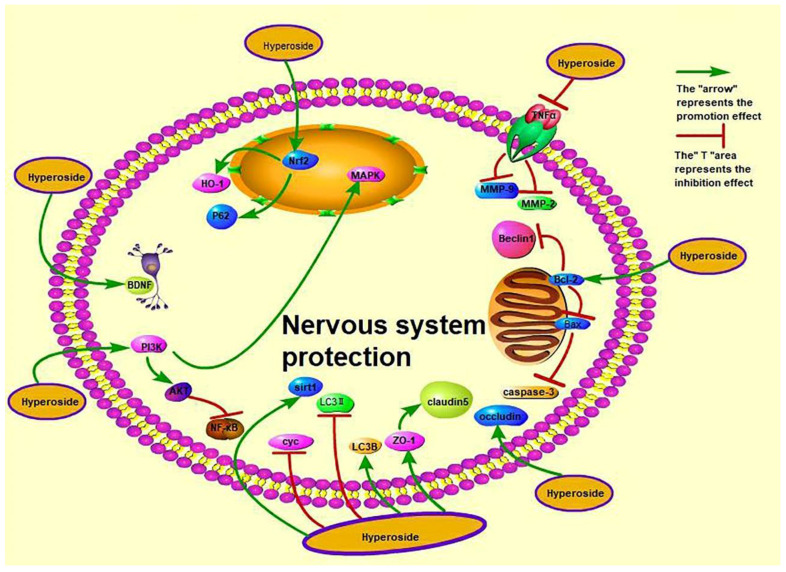
Nerve-protective effect of hyperoside.

Nervous system diseases occur in the central nervous system, peripheral nervous system and vegetative nervous system, with sensory, motor, consciousness and vegetative nervous system dysfunctions as the main manifestations of disease. Nervous system diseases include depression, epilepsy, Huntington’s disease, neurodegenerative diseases, and so on. Hyperoside exerts its anti-depressant effects possibly through the serotoninergic system, monoaminergic system and BDNF up-regulation [38,46]. In contrast, it exerts its anti-epileptic effect by increasing the antioxidant level and reducing the levels of autophagy-related proteins through the PI3K/AKT and MAPK pathways [39]. Systemic degenerative diseases, including Alzheimer’s disease and Parkinson’s disease (PD), are primary degenerative diseases of the central nervous system caused by the deposition of extracellular β-amyloid protein (amyloid-β, Aβ). Liu et al. showed that hyperoside dose-dependently up-regulates zonula occludens-1 (ZO-1), occludin and claudin-5 and down-regulates MMP (matrix metalloproteinase)-2 and MMP-9 to protect the damaged or weakened blood–brain barrier (BBB) [45]. However, the pathogenesis of Alzheimer’s disease is diverse. Damaged or weakened BBB protection is only one mechanism in the pathogenesis of Alzheimer’s disease, and the authors only studied the mechanism in vitro. Therefore, more research is required on the specific effects of hyperoside on Alzheimer’s disease in vivo and its other pathogenetic pathways to enrich this field. Previous studies found that hyperoside can reduce the expression of caspase3, Cyc and Bcl-2, induce HO-1 activation of Nrf2 and inhibit 6-hydroxydopamine (6-OHDA)-induced oxidative stress to prevent and treat Parkinson’s disease [41,44]. Kwon et al. were the first to study the neuroprotective effect of hyperoside on 6-OHDA-induced neurotoxicity and its possible mechanism. This study promotes the application of hyperoside in the treatment of diseases related to Parkinson’s disease. Hyperoside can also reduce neuroinflammation, cognitive impairment and oxidative stress in type 2 diabetic rats through the tumour necrosis factor-α (TNF-α)/NF-κB/caspase-3 signalling pathway, activate the *SIRT1* gene and inhibit the nuclear factor-kappa-gene binding (NF-κB) gene to protect human neuroblastoma cells (SH-SY5Y) from oxidative damage [37,40]. Furthermore, hyperoside can protect the nerves of mice from cerebral ischemia–reperfusion injury. Intragastric administration of 50 mg/kg hyperoside increases the expression of ZO-1 and Claudin5 protein in mice [35]. In addition, intragastric administration of hyperoside to rats exerts significant anti-depressant-like effects (1.8 mg/kg/day p.o.) (*p* < 0.05) [122]. For example, intraperitoneal injection of hyperoside 1 mg/kg and 10 mg/kg shortened the immobile time of rats to 78.92 ± 3.32 and 69.33 ± 4.7 s (*p* < 0.05) and increased sucrose consumption by 103% ± 7.22% and 128% ± 11.1%, respectively (*p* < 0.01) [123]. Treatment with hyperoside (0.6 mg/kg/day) for 2 weeks significantly reduces plasma adrenocorticotropic hormone and corticosterone levels by 40%–70% [124]. However, current research on hyperoside still faces several problems. For example, the research on the prevention and treatment of neurological diseases by using hyperoside is still in the experimental stage, and few clinical studies have been conducted. A consensus on a safe and effective dose of hyperoside for the human body has yet to be reached, and the clinical efficacy of the treatment is affected by many factors. Therefore, the clinical value and effective concentration of hyperoside needs to be explored and studied further. In addition, Huntington’s disease is a neurological disease, but scholars have yet to study whether hyperoside exerts a therapeutic effect on this disease. The mechanisms by which hyperoside exerts nerve protection are shown in Figure 4.

### 3.10. Cardioprotective Activity

**Figure 5:**
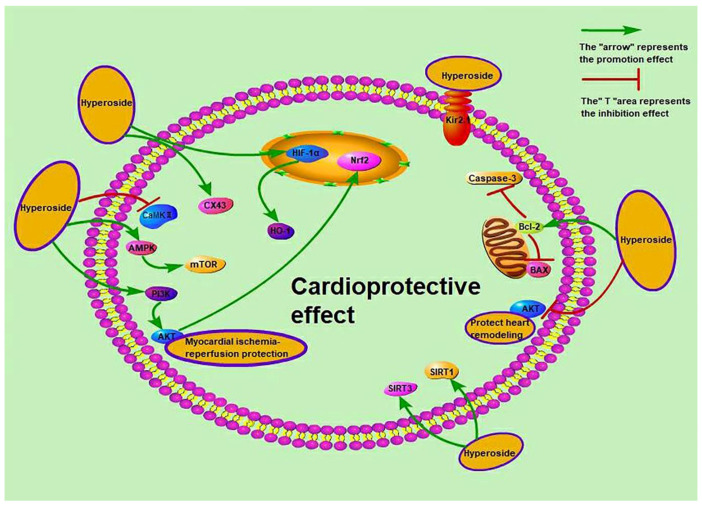
Cardioprotective activity of hyperoside.

Cardiovascular diseases, including myocardial hypertrophy, atrial fibrillation, heart failure and myocardial ischaemia–reperfusion, are commonly caused by abnormal heart function or structural defects. Hyperoside blocks the AKT pathway, which reduces the protein expression of B-type natriuretic peptide and β-myosin heavy chain by angiotensin II (Ang II) or enhances SIRT3 signal expression to improve cardiac hypertrophy [55,59]. Hyperoside also protects against myocardial ischaemia and reperfusion. The activated related pathways are protein kinase 1 (PKC)/mitochondrial ATP channel (mitoKATP) and AMPK/mTOR, and the affected proteins are gap junction protein 43 (Cx43), inwardly-rectifying potassium channel 2.1 (Kir2.1) and calmodulin kinase II (CaMKII), which also affects the activity of myocardial ATPase [47,50,54,56]. In addition, myocardial infarction in mice and heart failure in rats are related to the regulation of autophagy [53,57,60], and myocardial infarction is also related to the nucleotide binding oligomerization domain like receptor 1 (NLRP1) inflammatory pathway [60]. Hyperoside protects the myocardium of severely burned rats by regulating inflammation and oxidative stress and activating the SIRT1 signalling pathway [51]. At the same time, hyperoside can treat sepsis-related cardiac dysfunction by inducing the hypoxia-inducible factor-1α (HIF-1α)/HO-1 signalling pathway or inhibiting microRNA-21 (miR-21) [52,61]. Up-regulation of microRNA-138 (miR-138) can protect cardiomyocytes induced by hypoxia [58]. Hyperoside also protects the myocardial damage caused by diabetes and high glucose [48,49]. The mechanisms of cardioprotective activity are presented in Figure 5. Other research has shown that gavage of hyperoside (20 mg/kg/day) increases the left ventricular ejection fraction to 40.8% ± 5.1%, increases dp/dt max to 8735.4 ± 478.4 mmHg/s and decreases dp/dt min to −7902.3 ± 369.3 mmHg/s. It also decreases heart size and cardiomyocyte cross-sectional area [59]. Intraperitoneal injection of 50 mg/kg hyperoside decreases the infarct size in rats from 48.35 ± 6.74 to 23.61 ± 4.29 (*p* < 0.01) [125]. Hyperoside can ameliorate heart failure induced by thoracic aortic coarctation in rats and reduce myocardial cell cross-sectional area and heart weight/body weight ratio [57]. These studies suggest that hyperoside prevents stress overload-induced cardiac remodelling, alleviates myocardial ischemia–reperfusion injury and prevents heart failure, among others. However, whether hyperoside affects atrial fibrillation has not been studied.

### 3.11. Hepatoprotective Activity

Liver-related diseases include fibrosis, non-alcoholic fatty liver disease, and so on. Hyperoside can activate the Nrf2 gene to protect the acute liver injury induced by N-acetyl-para-amino-phenol [64]. Hyperoside also exerts a protective effect on acute liver injury induced by CCl4 via two mechanisms. One is to increase the Nrf2 level by increasing extracellular signal-regulated protein kinase 1/2 (ERK1/2)-chromosomal region maintenance 1 (Crm1), thereby protecting the liver from injury induced by CCl4 [62]. The other is to regulate the pleckstrin homology domain leucine-rich repeat protein phosphatase 2 (PHLPP2)-AKT-GSK-3β signalling pathway and reverse the decrease in SOD activity in the body [67]. At the same time, the factor member 1 of nuclear receptor subfamily group 4A (Nr4A1) related to Nrf2 is linked to the prevention of non-alcoholic fatty liver disease by hyperoside [66]. Hyperoside exerts a hepatoprotective effect on diabetic mice, rats with liver fibrosis caused by heart failure and rats with hepatic ischaemia–reperfusion injury [63,65,68]. In rats, hyperoside (15 and 60 mg/kg) induces the reversal of serum alanine aminotransferase and aspartate transaminase levels and protects liver tissue from CCl4-induced injury [126]. Intraperitoneal injection of hyperoside (50 mg/kg/day) decreases the Suzuki score of the liver from 6.0 ± 0.9 to 5.0 ± 0.5 (*p* < 0.05) and histological damage [65]. In addition, gavage of 100 and 200 mg/kg hyperoside improves vacuolar oedema and degeneration of liver cells and inhibits alanine aminotransferase, aspartate aminotransferase and alkaline phosphatase levels in rats [63]. These results reflect the protective effect of hyperoside on the liver, though there is a need for researchers to study the effect of hyperoside on alcoholic fatty liver disease.

### 3.12. Renal Protective Activity

Kidney disease has risen from the 13th to the 10th leading cause of death worldwide. The death rate increased from 813,000 in 2000 to 1.3 million in 2019 (https://www.who.int/cancer) (accessed on 10 January 2022). Hyperoside can improve diabetic nephropathy by targeting the miR-499-5p/APC axis and inhibiting the extracellular regulated kinase (ERK)/cAMP-response element binding protein (CREB)/miRNA-34a signalling pathway [79,80]. It can also treat acute kidney injury by regulating mitochondrial fission mediated by metalloproteinase-associated protein 1 (OMA1)-optic atrophy 1 and inhibiting TLR4 and nucleotide binding oligomerisation domain-like receptorprotein3 (NLRP3) pathways [74,78]. Liu et al. reported that hyperoside can inhibit autophagy through the AMPK-unc-51 like autophagy activated kinase 1 (ULK1) signalling pathway to prevent age-related renal injury, and provided the first opportunity for hyperoside to treat D-galactose-induced renal aging and damage [77]. In addition to the above three aspects, hyperoside can also improve the endogenous antioxidant and detoxification functions of kidney cells through the Nrf2/HO-1/quinone oxidoreductase 1 (NQO1) pathway [76]. Intragastric administration of hyperoside and quercetin (20 mg/kg/day) at a ratio of 1:1 can reduce the severity of renal crystal deposition (*p* < 0.05) [127] and reduce urinary citrate excretion to 48.38 ± 22.82 mg/24 h [127]. This finding suggests that hyperoside prevents calculi in rats. A mixture of quercetin and hyperoside (0.1 mg/kg/day) (1:1) was administered intragastrically to rats to reduce the expression of fibrosis-related proteins in obstructed kidneys [128], thereby protecting the kidney. Interestingly, although both experiments proved that hyperoside and quercetin (1:1) exert a protective effect on kidney diseases, the difference in dose gap is very large; thus, the accurate dosage could be explored in the future to provide a preliminary basis for clinical trials.

### 3.13. Protective Activity of Bone and Joint Diseases

Hyperoside has a protective effect on interleukin-1β (IL-1β)-induced osteoarthritis and rheumatoid arthritis. The pathways related to its protective mechanism include the NF-κB signalling pathway, p38 protein kinase pathway, PI3K/AKT/NF-κB and MAPK signalling pathway [88,92,95]. The nuclear factor receptor activator κB ligand (RANKL)/RANK/NF-κB signalling pathway is also related to the NF-κB signalling pathway. Experiments have verified that inhibiting the RANKL/nuclear factor kappa B receptor activator (RANK)/NF-κB signalling pathway can improve osteoporosis in ovariectomised (OVX) mice [89]. In addition, the factors used to treat osteoarthritis include TNF-α, IL-6, MMP3 and MMP13 [93,94]. Inhibiting the MAPK signalling pathway and regulating the TWEEP-p38 pathway also contribute to the protective effect of hyperoside on osteoblasts [90,91].

### 3.14. Others

Firstly, hyperoside can protect blood vessels. Hyperoside can reduce the production rate of ArgII by competing for active sites, changing the surface hydrophobicity of the enzyme, decreasing the vascular tone and inhibiting vascular remodelling, thereby lowering blood pressure [86]. Liu et al. used network pharmacology to elucidate the mechanism of anti-atherosclerosis treatment, which may be mainly related to the PI3K/AKT and MAPK signalling pathways [129]. Other researchers have demonstrated that hyperoside can regulate vascular endothelial cells by reducing low-density lipoprotein-C level, affecting nitric oxide synthase (NOS) activity, regulating NO synthesis, improving vascular endothelial function and reducing p38 MAPK, Jun N-terminal kinase (JNK), ERK, NF-κB and TNF receptor 1 (TNFR1) levels to inhibit vascular inflammation and affect atherosclerosis [85,87]. By contrast, Wang et al. found that hyperoside cannot reduce blood lipids in mice and cannot inhibit the formation of aortic atherosclerotic plaques [130]. Secondly, hyperoside also exhibits lung-protective functions. Hyperoside protects bleomycin-induced pulmonary fibrosis through the AKT/GSK3β pathway and inhibits collagen secretion [81,83]. It also inhibits AMPK/mTOR signalling to reduce particulate-induced lung injury [82], and can be used to treat *Mycoplasma pneumoniae* pneumonia (MPP) via the interaction of chemokine ligand 5 (CCL5)-CC chemokine receptor 4 (CCR4) [84]. Thirdly, hyperoside has a function in ovarian protection. The protection of hyperoside in ovarian-related diseases is related to SHH signalling pathway, the PI3K/AKT anti-apoptotic pathway and Nrf-2/HO-1 anti-oxidative stress [97,98]. Fourth, hyperoside has an anti-inflammatory effect [96,102]. This anti-inflammatory effect is related to the miR-499a-5p/nuclear receptor interaction protein 1 (NRIP1) axis, regulation of the p38MAPK/Sirt6/NF-κB signalling pathway and inhibition of the TLR4/NF-κB pathway [99,103,104]. Hyperoside (100 mg/kg, i.p. (*p* < 0.05) and 200, 500 mg/kg, p.o. (*p* < 0.01)) significantly inhibits acetic acid-induced vascular permeability in mice [131]. In addition, 100 mg/kg hyperoside significantly decreases serum prostaglandin E2 (PGE2), TNF-α, IL-1β, c-reactive protein (CRP), myeloperoxidase (MPO) and MDA levels (*p* < 0.01) and significantly increases SOD activity in mice (*p* < 0.01) [132]. Haematoxylin and eosin results proved the effect of hyperoside on ulcerative colitis, suggesting that hyperoside demonstrates good anti-inflammatory activity. Intragastric administration of 100 mg/kg hyperoside in rats significantly reverses the up-regulation of N-methyl-d-aspartic acid (NMDA) receptor containing n-methyl-d-aspartate receptor 2B (NR2B) in the midbrain periaqueductal grey and shows analgesic activity against continuous inflammatory stimulation in mice [133]. Hyperoside can also prevent age-related macular degeneration and protect against diabetic retinopathy [100,101]. It also has antioxidant activity [134,135,136,137]. Wang Mengyu reported that the antioxidant activity of hyperoside is related to the 3-position hydroxyl group of hyperoside [138]. Hyperoside can also protect the pancreas, fight fatigue and enhance NK cell proliferation [105,107,108]. In addition, hyperoside regulates the mTOR/S6K and TLR4/myeloid differentiation factor 88/NF-κB signalling pathways to reduce recurrent pregnancy loss and anterior cruciate ligament injury [109,110]. Pan Shanshan used a multi-omics strategy to demonstrate that hyperoside can regulate the metabolism of high-fat mice by changing the abundance of intestinal flora and down-regulating the expression of Cypla2 and Ugtla6b [137].

## 4. Pharmacokinetics

Hyperoside has a wide range of pharmacological effects and pharmacokinetic characteristics, such as easy accumulation in the viscera and kidneys, low oral bioavailability and compatibility with different drugs that prolong its elimination time in the body. The pharmacokinetics of hyperoside will be discussed in detail below. Ni et al. found that the extraction of hyperoside impurities from dodder seed by using ethyl acetate has minimal interference, high recovery and stability, and that using icariin as an internal standard could reduce errors in sample handling and injection; in addition, the hyperoside curve shows the main and secondary peaks [139]. Another scholar reported that the Cmax in rats intragastrically administered with Qianbai rhinitis capsules was 1.25 times that of rats treated with Senecio extract [140]. This result shows that hyperoside is compatible with other traditional Chinese medicines and they can improve its bioavailability and oral absorption. Chen et al. also found that t_1/2_, T_max_ and AUC_0-∞_ are significantly prolonged after hyperoside is combined with other Chinese medicines, indicating that they can slow down the elimination of hyperoside in vivo, prolong the action time, promote its absorption and significantly improve bioavailability. They also found the highest accumulation of hyperoside occurred in the kidney, followed by the liver and lastly in the testes [141]. Chen Shanshan also studied the pharmacokinetics of hyperoside when administered multiple times and showed that this treatment improves C_max_, T_max_, AUC_(0-T)_, AUC_(0-ꝏ)_ and MRT [142]. Yuan et al. studied the effects of different administration methods in rats and found that the plasma levels from intraperitoneally administered hyperoside are closer to those of intravenously administered hyperoside than to those of intragastrically administered hyperoside; in addition, the bioavailability of hyperoside in rats is particularly low after intragastric administration [143]. These results indicate that intraperitoneal and intravenous injections are effective ways of administration. The pharmacokinetic profile of hyperoside is presented in Table 12.

## 5. Toxicity

Hyperoside has many pharmacological effects, including significant renal protection. Previous pharmacokinetic studies indicated that hyperoside accumulates in the kidney. However, studies on the toxicity of hyperoside are very few. So far, only one team has studied the toxicity of hyperoside, and only animals were used in their studies. Firstly, an acute toxicity test of hyperoside showed that its LD50 > 5000 mg/kg [145]. Secondly, a bacterial reverse mutation assay (Ames test) indicated that hyperoside has no genetic toxicity [145]. An experiment on rat embryo and foetal developmental showed that this compound exerts negligible effects on pregnant rats but slows down the growth of foetal rats [146]. Thirdly, long-term use of hyperoside is toxic to the kidneys, but the damage is reversible [147]. However, research on the toxicity of hyperoside is not comprehensive, and a cellular experiments that verify whether or not hyperoside is cytotoxic remain to be conducted. Therefore, experiments must be conducted in the future to evaluate the biological safety of hyperoside and provide a basis for its future clinical applications.

## 6. Conclusions and Perspective

At present, many studies have shown that hyperoside can be found in Hypericaceae, Rosaceae and Polygonaceae plants. However, the plant family with the highest abundance of hyperoside cannot be determined because of the different measurement conditions used. Hyperoside has anti-cancer, brain-protective, neuroprotective, cardioprotective and renal-protective activities, among others. However, most scholars have only studied classic signalling pathways, such as PI3K/AKT and NF-κB, and few scholars have studied other pathways. In the future, scholars could concentrate on different pathways to study the effects of hyperoside on target diseases to promote the advancement of medicine worldwide. At present, few studies have explored the pharmacokinetics, especially the excretion, of hyperoside. However, by consulting the existing literature on the pharmacokinetics of hyperoside, we can conclude that the drug-time curve after oral administration of hyperoside in rats shows bimodal absorption. This phenomenon may be related to hepato-enteric circulation or absorption by dual parts of the intestine, though these conjectures have not been confirmed by researchers. Studies have also shown that the bioavailability of orally administered hyperoside is lower than that of intraperitoneally injected hyperoside, which may be related to the first-pass metabolism of hyperoside and the physical properties of flavonoids (hydrophobicity). In response, researchers have developed hyperoside–zein/pectin composite nanoparticles and hyperoside-loaded zein–tea polyphenols–pectin ternary complex nanoparticles to slow the release of hyperoside [14,15]. This system improves the bioavailability of hyperoside. In addition, other scholars have found that combining hyperoside with other drugs can slow down its elimination in the body and prolong its action time, thereby increasing its bioavailability. Hyperoside is also used to treat chronic diseases, such as atherosclerosis, but its safety remains to be verified. As a result, the clinical application of hyperoside is limited. In short, the research on the pharmacology and pharmacokinetics of hyperoside is insufficient, which directly restricts further therapeutic development of hyperoside. This review has summarised the pharmacology and pharmacokinetics of hyperoside and raised some issues worthy of future discussion to promote the application and development of hyperoside in the future.

## Figures and Tables

**Table 1:** Anti-cancer Activity of Hyperoside.

Detail	Cell Lines/Model	Dose	Ref.
Anti-cancer activity			
Up-regulates caspase-3, caspase-8, Bax, p53 and MDA contents; decreases GSH, SOD and CAT activities; decreases VEGF and Bcl-2 levels; and inhibits cell growth.	HeLa	100 μmol/L	[20]
Down-regulates Nampt, NAD and Sirt1 mRNA and protein expression and inhibits cell proliferation and migration.	HeLa	400 μg/mL	[21]
Inhibits cell proliferation in a dose- and time-dependent manner.	Cervical cancer HeLa and C-33A cells	0.25, 0.5, 1, 2, 4 and 8 mM	[22]
Up-regulates p53, caspase-9 and caspase-3 expression and inhibits cell proliferation and apoptosis.	Human hepatoma HepG2	20 and 50 nmol/L	[23]
Down-regulates BMP-7 expression, AKT phosphorylation and PI3K expression; induces cell cycle arrest; and inhibits cell proliferation.	Human HepG2	5, 10, 20, 40 and 80 μM	[6]
Down-regulates PD-L1 and the protein expression of transcription factor c-Myc and prevents tumour formation.	Human non-small cell lung cancer H1975 and HCC827	0, 2, 5 and 10 μmol/L	[24]
Decreases cell hypoxia-induced survival and proliferation; up-regulates AMPK phosphorylation and HO-1 expression; and inhibits cell survival and proliferation.	Human NSCLC	10, 50 and 100 μM	[25]
Inhibits proliferation, induces apoptosis and up-regulates FoxO1 expression.	Adenocarcinoma lung cancer PC-9 and T790M-positive NSCLC	150 µM	[26]
Induces apoptosis and G1/S phase arrest and inhibits cell proliferation.	Human lung adenocarcinoma A549	100 μg/mL	[27]
Inhibits cell viability and migration; increases cell apoptosis; down-regulates Bcl-2 and X-linked inhibitor of apoptosis; and up-regulates Bax and cleaved caspase-3.	4T1 and MCF-7	25, 50 or 100µM	[28]
Down-regulates Bcl-2; up-regulates Bax and IL-6; may elevate cell sensitivity to paclitaxel.	Human normal breast epithelial cell line MCF-10A and breast cancer cell lines	5, 10, 50 and 100 μg/mL	[29]
Up-regulates caspase-3, Bax and IκBα expression; down-regulates NF-κB, P65 and Bcl-2 expression; induces cell apoptosis; and blocks cell cycle in the G0/G1 phase.	Human gastric cancer MKN-45	50 and 100 μg/mL	[30]
Reduces cell proliferation; down-regulates P65 and Bcl-2 expression; and up-regulates caspase-3 and IκBα expression.	human gastric cancer MKN-45	50, 75 and 100 µg/mL	[31]
Inhibits SKOV3 cell proliferation; promotes cell apoptosis; up-regulates cleaved caspase-3 and caspase-9; down-regulates Bcl-2, p65 and p-IκB-α; and decreases cell migration and invasion.	Human ovarian cancer SKOV3	5, 15 and 25 μmol/L	[32]
Up-regulates perforin and granzyme B expression and increases killing activity of NK cells against PANC 1 cells.	Human NK cells and pancreatic cancer PANC1	1.6 and 8 μg/mL	[33]
Reduces the changes in phosphorylated levels of PI3K, AKT, mTOR and AMPK while decreasing p38 phosphorylation; inhibits proliferation; and induces apoptosis and autophagy. It may have a therapeutic value in the chemoprophylaxis of skin cancer.	Skin cancer cell lines and DMBA/TPA-induced skin tumours	25 and 50 μM	[34]

Notes: Nampt, nicotinamide phosphoribosyltransferase; NAD, nicotinamide adenine dinucleotide; Sirt1, silent information regulator 1; caspase, cysteinyl aspartate specific proteinase; Bax, Bcl-2 associated x protein; p53, tumour suppressor gene; MDA, malondialdehyde; GSH, glutathione; SOD, superoxide dismutase; CAT, catalase; VEGF, vascular endothelial growth factor; Bcl-2, B cell lymphoma-2; BMP-7, bone morphogenetic protein 7; AKT, protein kinase B; PI3K, phosphoinositide 3-kinase; PD-L1, programmed death ligand 1; c-Myc, proto-oncogene protein c-Myc; MAPK, mitogen-activated protein kinase; HO-1, haem oxygenase-1; FoxO1, forkhead box protein O1; NSCLC, non-small cell lung cancer; IL, interleukin; mTOR, mammalian target of rapamycin; AMPK, 5′AMP-activated protein kinase; NF-κB, nuclear factor kappa-B.

**Table 2:** Neuroprotective Activity of Hyperoside.

Detail	Cell Lines/Model	Dose	Ref.
Increases the number of NeuN-positive cells; decreases the number of GFAP- and PECAM-positive cells; up-regulates ZO-1 and claudin5 protein expression; maintains the integrity of the blood–brain barrier; and may protect neural function in CIR-injured mice.	CIR injury induced by MCAO in mice	50 mg/kg	[35]
Inhibits the activation of microglia and the synthesis of inflammatory factors after SAH; increases the phosphorylation of AKT and GSK-3β; alleviates early brain injury after subarachnoid haemorrhage; and promotes nerve function recovery in rats.	SAH in rats	50 mg/kg	[36]
Increases cell survival rate; decreases LDH release; reduces cleared ROS level, MDA content and caspase-3 activity; increases SOD and CAT activities and GSH content; increases SIRT1 gene expression; down-regulates NF-κB mRNA and protein expression; and protects against high glucose-induced oxidative damage of human neuroblastoma cells.	SH-SY5Y	50 and 100 μm L/L	[37]
Significantly shortens the cumulative immobility time of forced swimming and tail suspension mice; can act as an anti-depressant.	Depressive ICR mice	20 mg/kg	[38]
Targets the PI3K/AKT and MAPK pathways to increase antioxidant levels and decrease the levels of autophagy-related proteins; protects the hippocampal CA3 region from neuronal damage caused by epilepsy.	Neuronal damage in a mouse model	50 mg/kg	[39]
Decreases TC, TG and LDL-C levels and prevents cognitive dysfunction, neuroinflammation and oxidative stress in diabetic rats.	Type 2 diabetes rats	50, 200 and 400 mg/kg/day	[40]
Reverses the regulation of Beclin1, LC3, Bax, cleaved caspase-3, Cyc and Bcl-2 expression in rat SNpc tissues and SH-SY5Y cells; promotes the regulation of P62 and α-synuclcin; and exerts a neuroprotective effect.	Rotenone was used to induce Parkinson’s disease rat model and SH-SY5Y cell injury model	100 and 200 mg/kg 0.5, 1 and 2 μmol/L	[41]
Activates the PI3K, AKT and LC3B pathways; deactivates the NF-κB, Bax, caspase 3 and P62 pathways; and inhibits oxidative stress damage.	Hyperoside on rat pheochromocytoma (PC12) cells	12.5, 25 and 50 μmol/L	[42]
Down-regulates IL-1β, IL-6, IL-8, TNF-α, ROS, MDA, Bax and caspase-3 levels; increases CAT, SOD and GSH activities; up-regulates Bcl-2, BDNF, TrkB, SIRT1 and NGF expression; reduces LPS-induced inflammation, oxidative stress and apoptosis; and protects against neuroinflammation.	HT22	20 μM	[43]
Significantly improves neuronal cell viability loss, lactate dehydrogenase release, excessive ROS accumulation and mitochondrial membrane potential dysfunction and inhibits neuronal death.	Human dopaminergic neuroblastoma SH-SY5Y	0.5, 1 and 2 µM	[44]
Decreases Bax/Bcl-2 ratio, down-regulates cytochrome c expression and caspase-3 activity, up-regulates zonula occludens-1 and claudin-5 expression; and minimises damage to the blood–brain barrier.	Fibrillar Aβ1–42-induced disruption in an in vitro blood-brain barrier model	200 or 500 µM	[45]
Decreases the immobility of prefrontal lobe and caudate putamen and exerts anti-depressant effects.	Forced swimming test and tail suspension test in mice	1.875, 3.75 and 7.5 mg/kg	[46]

Notes: Aβ1–42, amyloid beta 1–42; CIR, cerebral ischaemia–reperfusion; LC3B, light chain 3 beta; MCAO, middle cerebral artery embolism; SAH, subarachnoid haemorrhage; NeuN, counting neuronal nuclear antigens; GFAP, glial fibrillary acidic protein; PECAM, platelet endothelial cell adhesion molecule; ZO-1,: zonula occludens-1; LDH, lactate dehydrogenase; ROS, reactive oxygen species; CAT, catalase; NF-κB, transcription factor kappaB; SH-SY5Y, human neuroblastoma; TC, total cholesterol; TG, triglycerides; LDL-C, low-density lipoprotein-C; LC3, autophagy marker; HT22, mouse hippocampal neurons; TNF, tumour necrosis factor; BDNF, brain-derived neuro-trophic factor; TrkB, tyrosine receptor kinase B; NGF, nerve growth factor; LPS, lipopolysaccharide.

**Table 3:** Cardioprotective Activity of Hyperoside.

Detail	Cell Lines/Model	Dose	Ref.
Decreases the levels of AST, CK, CK-MB and c-TnT in rats; the rate of cardiomyocyte apoptosis; and the relative expression of protein and CaMK II protein. Effectively relieves heart failure.	Rat model of myocardial ischaemia–reperfusion injury	50,100 mg/kg	[47]
Decreases fasting blood glucose, cTnI I and MDA contents and increases SOD activity, the value of ejection fraction and short-axis shortened pitch in diabetic mice; improves glycolipid metabolism; and exerts a protective effect on myocardial injury caused by diabetes.	Type 2 diabetic mice induced by high-fat diet combined with low-dose streptozotocin	ND	[48]
Increases cell survival rate; decreases cell apoptosis rate and ROS and MDA levels; increases SOD level, PI3K relative expression level and AKT and Nrf2 phosphorylation level; and protects cardiac muscle cells from high sugar-induced oxidative stress damage.	High-glucose treatment simulates oxidative stress injury of cardiomyocytes	4, 8 and 20 nmol/L	[49]
Decreases MDA content and CK-MB activity; increases SOD activity and ATP content; up-regulates Nrf2, PKCε and Kir6.2 expression in cardiomyocytes; and improves cardiac muscle damage.	Myocardial I/R injury in rats	50 mg/kg	[50]
Decreases CK-MB, TNF-α and IL-1β expression in serum; increases SOD and GSH activities in myocardial tissue; up-regulates SIRT1 protein expression; down-regulates FoxO1 acetylation level and apoptosis protein cleaved caspase-3 expression; alleviates early myocardial injury caused by antioxidative stress and anti-inflammatory effects in severely burned rats.	Early myocardial injury in severely burned rats	30 mg/kg	[51]
Increases the levels of LVSP, maximum increase rate of left ventricular pressure (+dp/dt_max_), maximum decrease rate of left ventricular pressure (−dp/dt_max_), HIF-1α and HO-1; decreases the levels of LVEDP, cTnI, BNP, TNF-α and IL-1β; and ameliorates myocardial injury in septic rats.	Myocardial cells of sepsis model in rats	10 and 20 mg/kg	[52]
Decreases serum oxidative stress; improves thoracic aorta remodelling and endothelial dysfunction to a certain extent; exerts a cardiovascular protective effect on mice with myocardial infarction.	Myocardial infarction model	36 mg/kg	[53]
Increases the levels of heart rate, mean arterial pressure and heart rate systolic pressure product; decreases the levels of CK-MB and cTnI; and reduces I/R ventricular arrhythmia in rats.	Myocardial I/R model in rats	50 mg/kg	[54]
Significantly enhances SIRT3 signal expression; alleviates oxidative stress injury of myocardial tissue; inhibits the progression of myocardial fibrosis; and improves pathological myocardial hypertrophy caused by stress load.	Myocardial hypertrophy (mouse)	50 mg/kg/day	[55]
Increases phosphorylated AMPK, phosphorylated mTOR and P62 proteins; decreases apoptosis index, caspase-3 activity and LC3II and Beclin1 expression; and alleviates I/R injury of H9C2 cells.	I/R model	50 μmol/L	[56]
Decreases cardiac myocyte cross-sectional area and cardiac weight/body weight ratio; inhibits autophagy in TAC rats and AngII-induced H9C2 cells and apoptosis; and effectively alleviates heart failure by inhibiting apoptosis and inducing autophagy.	TAC-induced heart failure in rats	100 and 200 mg/kg	[57]
Decreases number of apoptotic cells; down-regulates lytic caspase-3 expression; up-regulates Bcl-2 expression; increases survival of myocardial cells; and alleviates hypoxic injury.	Hypoxia model in H9C2 cells and C57BL/6 mice	50 µmol/L, 50 mg/kg	[58]
Inhibits AngII-induced cardiomyocyte hypertrophy; protects against stress overload-induced cardiac remodelling;	Myocardial hypertrophy model in mice	20 mg/kg/day	[59]
reduces infarct CVF and myocardial hypertrophy; exerts obvious protective effect on heart injury in mice with myocardial infarction.	Myocardial mouse model	18 and 36 mg/kg	[60]
Decreases cell vitality and aggravates inflammation; down-regulates miR-21 expression in cardiomyocytes; increases cell survival rate; decreases inflammatory response; inhibits miR-21; and regulates cardiomyocyte activity and inflammation.	Sepsis mouse model, and myocardial injury cell model	20 mg/kg, 10 μM	[61]

Notes: AngII, angiotensin II; CaMK II, calmodulin-dependent protein kinase II; CK, creatine kinase; CK-MB, creatine kinase isoenzyme; cTnI, troponin; c-TnT, cardiac troponin T; CVF, collagen volume fraction; I/R, ischaemia–reperfusion; LVEDP, left ventricular end-diastolic pressure; LVSP, left ventricular systolic pressure; TAC, thoracic aortic constriction; ND, not determined; AST, aspartate aminotransferase; Nrf2, nuclear factor E2-related factor2; ATP, adenosine triphosphate; PKCε, Protein kinase Cε; TNF, tumour necrosis factor; HIF-1α, hypoxia-inducible factor-1α; BNP, B-type natriuretic peptide.

**Table 4:** Hepatoprotective Activity of Hyperoside.

Detail	Cell Lines/Model	Dose	Ref.
Decreases liver index, AST, ALT, MDA and Bach1 complex levels and alleviates the pathological damage of acute liver injury mice. Increases SOD and GSH activities and Nrf2–complex level; up-regulates transporter CRM1 expression; enhances ERK1/2 activity; and reduces cell oxidative stress damage.	Acute liver injury caused by CCl4 Human hepatocytes L02 cells	100 mg/kg 100 and 200 μM	[62]
Decreases ALT, AST and ALP levels; down-regulates α-SMA, type I collagen, fibrotic factor-CTGF, MMP2 and MMP9; inhibits the activation of the transforming growth factor-β-1/Smad pathway and hepatic stellate cells mediated by transforming growth factor-β1; and prevents liver fibrosis.	A model of via aortocaval fistula in rats Hepatic stellate cell line LX-2	200 mg/kg 2 mM	[63]
Decreases AST, ALT and ALP levels; activates Nrf2 and its downstream genes; decreases SOD, GST and GSH-Px activities; reduces LPO, LDH and ALT production; and prevents acute liver injury.	N-APAP acute hepatic injury LO2 cells	100 mg/kg 20 μM	[64]
Decreases AST/ALT and MDA activities; increases SOD and glutathione peroxidase activities and haem oxygenase 1 and NAD(P)H expression of quinone oxidoreductase 1; down-regulates caspase-3 expression; and prevents hepatic ischaemia–reperfusion injury in rats.	70% hepatic ischaemia–reperfusion injury model	50 mg/kg/day	[65]
Up-regulates Nr4A1; improves liver steatosis, insulin resistance and inflammation; and may prevent the pathological progression of non-alcoholic fatty liver disease.	NAFLD Primary hepatocytes	50 mg/kg 1, 5 and 10 μM	[66]
Increases SOD activity and MDA level in the body; down-regulates PHLPP2 expression; activates AKT phosphorylation; induces GSK-3 double phosphorylation; and protects against oxidative stress-induced liver damage.	Carbon tetrachloride damages rat liver Human hepatocytes (L02 cells)	30 and 15 mg/kg 10 and 100 μM	[67]
Decreases total cholesterol, triglycerides and low-density lipoprotein cholesterol levels; inhibits the phosphorylation of p65/NF-κB and mitogen-activated protein kinase; activates transcription factor 3 protein expression; and decreases Bax, cytochrome c, caspase-9 and caspase-3 expression. It may be beneficial in the treatment of diabetes.	Diabetic mice induced by high-sugar and high-fat diet and alloxan	50,100 and 200 mg/kg bw/day	[68]

Notes: α-SMA, α-smooth muscle actin; ALP, alkaline phosphatase; ALT, alanine aminotransferase; CTGF, connective tissue growth factor; AST, serum aspartate aminotransferase; MMP, matrix metalloproteinase; Bach, BTB-CNC homolog 1; CRM1, chromosome maintenance protein 1; ERK, extracellular regulated kinase; Smad, drosophila mothers against decapentaplegic protein; LPO, lipid peroxidation; APAP, N-acetyl-para-amino-phenol; NAD(P)H, triphosphopyridine nucleotide; NAFLD, non-alcoholic fatty liver disease; PHLPP2, domain leucine-rich repeat protein phosphatase 2; GSK, glycogen synthase kinase.

**Table 5:** Brain-Protective Activity of Hyperoside.

Detail	Cell Lines/Model	Dose	Ref.
Down-regulates TNF-α, IL-1β, IL-6, ICAM-1, VCAM-1, TLR4, COX-2, NF-κB, caspase-3, caspase-9, Bax and Bcl-2 expression and prevents CIR injury.	Middle cerebral artery occlusion/reperfusion rat model	60 and 120 mg/kg	[69]
Decreases LDH activity and MDA, NSE and S100β contents; relaxes the cerebral basilar artery in a dose-dependent manner; up-regulates IP3, PKC, TRPV4, SKca and IKca expression; reduces Ca^2+^ fluorescence intensity; and ameliorates brain injury in rats with ischaemic stroke.	Global CIR model	50 mg/kg	[70]
Up-regulates BDNF expression in the hippocampus; down-regulates p75NRT; reduces hippocampal neuron and cell damage; improves learning and memory; and protects the hippocampal tissue in rats.	Middle cerebral artery occlusion, MCAO model	50 and 100 mg/kg	[71]
Up-regulates Bcl-2 mRNA, p-PI3K and p-AKT protein expression; down-regulates Bax and caspase-3 mRNA expression; and prevents hepatic IR injury in rats.	Cerebral I/R injury in rats	50 mg/kg/day	[72]
Increases SOD, catalase and glutathione peroxidase activities and improves cell apoptosis after nickel administration.	Nickel-induced brain damage in rats	50 mg/kg	[73]

Notes: ICAM, intercellular adhesion molecule; VCAM, vascular cell adhesion molecule; TLR, Toll-like receptor; COX, cyclo-oxygenase; NSE, neuron-specific enolase; S100β, serum central nervous system-specific protein; IP3, inositol trisphosphate; PKC, protein kinase 1; TRPV4, transient receptor potential vanilloid 4; SKca, small conductance; IKca, intermediate conductance Kca; CIR, cerebral ischaemia–reperfusion; BDNF, brain-derived neurotrophic factor; p75NRT, p75 neurotrophin receptor; MCAO, middle cerebral artery occlusion.

**Table 6:** Renal-Protective Activity of Hyperoside.

Detail	Cell Lines/Model	Dose	Ref.
Suppresses NLRP3, caspase-1 and ASC expression and prevents acute kidney injury induced by lipopolysaccharide.	Mouse acute kidney injury model	25, 50 and 100 mg/kg	[74]
Up-regulates Klotho protein expression; down-regulates P53, P21, IL-1, MCP-1, TGF-β, LC3 and Beclinl protein expression; decreases AMPK-ULK1 signalling pathway activity; prevents aging and damage of renal and tubular epithelial cells.	D-gal induces kidney aging and damage model	10 μg/mL 20 mg/kg/day	[75]
Decreases ROS and H_2_O_2_ levels and NADPH oxidase and LD activities and shows potential in the treatment of kidney stones and ROS-related diseases.	Human kidney-2 cells	100 and 200 µM	[76]
Up-regulates Klotho expression; down-regulates p53 expression; and prevents age-related kidney damage.	NRK-52E cells	5 and 10 μg/mL	[77]
Inhibits OPA1 hydrolysis, mitochondrial division, oxidative stress and apoptosis and shows new therapeutic potential in the treatment of acute kidney injury.	Renal ischaemia models	20 mg/kg	[78]
Decreases ERK pathway activation and downstream transcription factor CREB phosphorylation; down-regulates miRNA-34a expression; and inhibits high glucose-induced proliferation of mesangial cells.	Mouse glomerular mesangial cell line (SV40-MES13) diabetes model	50, 100 and 200 μM	[79]
Down-regulates APC expression; up-regulates miR-499e5p expression; and improves diabetic nephropathy by targeting the miR-499e5p/APC axis.	Mouse model of diabetic nephropathy	30 mg/kg	[80]

Notes: NLRP3, nucleotide binding oligomerisation domain-like receptorprotein3; ASC, apoptosis-associated speck-like protein; MCP, membrane cofactor protein; TGF, transforming growth factor; LC, microtubule associated protein I light; ULK1, Unc-51 like autophagy activated kinase 1; NADPH, nicotinamide adenine dinucleotide phosphate; LD, lactate dehydrogenase; NRK-52E cells, renal tubular duct epithelial cells in rats; OPA1, mediated proteolysis of optic atrophy 1; ERK, extracellular regulated kinase; CREB, cAMP-response element binding protein; APC, adenomatous polyposis coli.

**Table 7:** Lung-Protective Activity of Hyperoside.

Detail	Cell Lines/Model	Dose	Ref.
Decreases collagen I and III expression, serum TGF-β1 content and alveolar lavage fluid IL-6 levels and effectively improves bleomycin-induced pulmonary fibrosis in mice.	Pulmonary fibrosis model	100 mg/kg	[81]
Decreases cytotoxicity and p-AMPK expression; increases p-mTOR expression; inhibits the AMPK/α signalling pathway; down-regulates TNF-α and IL-6 expression in the alveolar lavage fluid; decreases total number of cells in the alveolar lavage fluid; and inhibits autophagy dysregulation and apoptosis by regulating the AMPK/mTOR pathway to prevent lung injury.	Human bronchial epithelial BEAS-2B	50 μM	[82]
Down-regulates MDA, TNF-α and IL-6 expression; increases SOD activity; inhibits epithelial–mesenchymal transition; and slows the development of pulmonary fibrosis by inhibiting oxidative stress and inflammation in the lung tissues of mice with pulmonary fibrosis.	Pulmonary fibrosis model	50 mg/kg/day	[83]
Increases the number of A549 cells; down-regulates IL-8 and TNF-α expression; and benefits *Mycoplasma pneumoniae* pneumonia through chemokine ligand 5–chemokine receptor 4 interaction.	Lung cancer A549	100, 200 and 400 µg/mL	[84]

**Table 8:** Vasoprotective Activity of Hyperoside.

Detail	Cell Lines/Model	Dose	Ref.
Reduces LDL-C, MDA and IL-6 levels; increases NO and eNOS levels; decreases plaque area in the aortic lumen of mice; improves lipid deposition; down-regulates PARP1, ARG2 and iNOS expression in mouse aortic tissue; and slows down atherosclerosis.	Atherosclerosis model	200 mg/kg	[85]
Decreases blood pressure and vascular tension, vascular remodelling, serum angiotensin-converting enzyme, ALD, U-mAlb, BUN, SCr, ALT and AST; increases fluorescence intensity of ANS-angiotensin-converting enzyme; inhibits vascular remodelling; and lowers blood pressure.	Rat hypertension model	23.2, 46.4 and 232.2 mg/kg	[86]
Decreases the adhesion of monocytes to TNF-α-stimulated VSMCs; down-regulates p38 MAPK, JNK and ERK, NF-κB and TNFR1 expression; inhibits vascular inflammation; and shows potential to prevent atherosclerosis.	Human monocyte U937 cell lines	1 and 10 µg/mL	[87]

Notes: INOS, nitric oxide synthase; eNOS, endothelial nitric oxide synthase; PARP1, poly(ADP-ribose) polymerase 1; ARG2, recombinant human arginase-2; ALD, aldosterone; U-mAlb, urine micro-albumin; BUN, blood urea nitrogen; SCr, serum creatinine; VSMCs, vascular smooth muscle cells; JNK, Jun N-terminal kinase; ERK, extracellular regulated kinase; TNFR1, tumour necrosis factor receptor 1.

**Table 9:** Bone-Protective Activity of Hyperoside.

Detail	Cell Lines/Model	Dose	Ref.
Increases IL-6 and TNF-α levels; decreases Col-I and Col-III levels; up-regulates MMP-3, MMP-9, p-IκB-α and p-p65 expression; prevents IL-1β-induced chondrocyte injury in the sacroiliac joint of mice; improves cell activity; and inhibits inflammatory factors and extracellular matrix disorder.	BALB/c mouse sacroiliac joint chondrocytes	50 and 100 μg/mL	[88]
Down-regulates RANKL, TRAF6 and IκBα expression; up-regulates NFATC1 and osteoprotegerin expression; and shows potential anti-osteoporotic effect on ovariectomised mice.	Ovariectomised mice	40 or 80 mg/kg/day	[89]
Increases ALP, Col-I and OCN mRNA expression; decreases the rate of apoptosis, expression of apoptosis-related proteins and levels of MC3T3-E1 phosphorylated JNK and p38; and protects osteoblasts by inhibiting MAPK signalling and oxidative damage of cells.	Osteoblastic MC3T3-E1 cells	20 and 40 μmol/L	[90]
Increases cell viability and proliferation; decreases cell apoptosis and autophagy; and protects osteoblasts from damage induced by Ti particles.	MC3T3-E1 cells	200 and 400 µg/mL	[91]

Notes: NFATC1, cytoplasmic nuclear factor 1; TRAF6, tumour necrosis factor receptor-related factor 6; Col-I, type I collagen; Col-III, type III collagen; MMP, matrix metalloproteinase; RANKL, receptor activator of NF-κB ligand; TRAF, tumour necrosis factor receptor-associated factor; NFATC1, recombinant nuclear factor of activated T-cells, cytoplasmic 1; IκBα, nuclear factor-κB α; OCN, osteocalcin; MC3T3-E1.

**Table 10:** Joint-Protective Activity of Hyperoside.

Detail	Cell Lines/Model	Dose	Ref.
Down-regulates INOS, MMP3 and p38 expression and inhibits the proliferation and migration of IL-1β-induced fibroblast synovial cells.	FLS cells	50 and 100 μm/mL	[92]
Decreases TNF-α and IL-6 contents in the serum; prevents the synovial hyperplasia and inflammatory cell infiltration of the mouse ankle joint; and exerts a certain therapeutic effect on rheumatoid arthritis.	CIA mice	25 and 50 mg/kg	[93]
Down-regulates MMP3 and TNF-a genes; reduces the loss of cell matrix of rat chondrocytes; maintains cell activity; down-regulates the expression of genes related to cell inflammation; reduces inflammation; and exerts a certain therapeutic effect on osteoarthritis in vitro.	SD rat chondrocyte osteoarthritis model	9 μg/mL	[94]
Down-regulates INOS, COX-2, MMPs and ADAMTS5; up-regulates type II collagen, agglutinin and SOX9; and exerts anti-arthritic effects.	Osteoarthritis model	20 and 40 μM 20 mg/kg	[95]

Notes: CIA, collagen-induced arthritis; FLS, fibroblast-like synovial; ADAMTS, recombinant A disintegrin and metalloproteinase with thrombospondin; SOX, Sry-related HMG box-containing gene 9.

**Table 11:** Other Effects of Hyperoside.

Detail	Cell Lines/Model	Dose	Ref.
Decreases IL-1β, TNF-α, MDA, MPO, NF-κB p65, TRAF6, LC3, Beclin1, p62 levels and cell apoptosis; increases SOD activity; and exerts obvious therapeutic effect on rat ulcerative colitis.	Ulcerative colitis	50 and 100 mg/kg	[96]
Increases serum E2, AMH, SOD and CAT activities; reduces FSH activity; up-regulates Nrf-2, HO-1, p-PI3K, p-AKT and Bcl-2 expression; decreases caspase3, Bax and ROS levels; improves tripterygium glycoside-induced primary ovarian insufficiency and mouse ovarian reserve function decline.	Adult endothelial cells	75 mg/(kg/day)	[97]
Decreases MDA content; increases SOD, GSH-Px and CAT activities; down-regulates Bax expression; up-regulates Bcl-2, SHH, Gli1 and SMO expression; and protects granular cells from H_2_O_2_-induced apoptosis and oxidative stress by activating the Sonic hedgehog signalling pathway.	Granular cells	40 μM	[98]
Increases MiR-499a-5p expression and decreases NRIP1 expression in a dose-dependent manner and mitigates apoptosis and inflammatory response induced by high glucose via the miR-499a-5p/NRIP1 axis.	HK-2 cells	10, 50 and 100 μmol/L	[99]
Up-regulates AhR target genes CYP1A1 and CYP1B1 and may prevent age-related macular degeneration.	Human retinal pigmented epithelial ARPE-19 cells and adenocaricinomic human alveolar basal epithelial A549 cells Animals exposed to UVA or blue light	25, 50 and 100 μM 25, 50 and 100 mg/kg	[100]
Down-regulates caspase-3, caspase-9 and Bax expression; up-regulates Bcl-2 expression; and possibly plays a protective role in diabetic retinopathy by reducing oxidative stress induced by high glucose and inhibiting cell damage and apoptosis.	Rat retinal vascular endothelial cells Rats fed with high-fat rat	10 mg/mL 20, 50 and 100 mg/kg	[101]
Increases the proliferation of rat bone mesenchymal stem cells and the number of EdU-positive cells; decreases cell cycle distribution; up-regulates Ki67 and PCNA expression; promotes the proliferation and osteogenic differentiation of bone marrow mesenchymal stem cells; and exerts potential therapeutic effect on periodontitis.	Bone marrow mesenchymal stem cells Rat periodontitis model	20 mL, 200 mg/mL	[102]
Down-regulates NO, TNF-α, IL-1β, IL-6 iNOS, p38 and NF-κB p65 expression; up-regulates Sirt6 expression; and inhibits the release of macrophage proinflammatory factors.	RAW264.7 macrophages	25, 50 and 100 μmol/L	[103]
Down-regulates IL-1β, IL-6, TNF-α and iNOS mRNA cleaved-caspase 8, 9, 3 and Bax expression; up-regulates Bcl-2 expression; and inhibits the inflammatory and apoptotic responses of human umbilical vein endothelial cells.	Human umbilical vein endothelial cells	20 and 50 μmol/L	[104]
Down-regulates TXNIP expression and intracellular calcium concentration; protects pancreatic beta cell function; and prevents type 2 diabetes and promotes its treatment.	Diabetic mice induced by high-fat diet and alloxan	200 and 400 mg/kg	[105]
Inhibits the expression of transcription factors and adipogenic genes and reduces lipid accumulation in adipocytes. Hyperoside at 5 µM inhibits adipogenesis, and hyperoside at 10 µM reduces fat accumulation in mature adipocytes.	3T3-L1 cells	10 μM	[106]
Decreases LA and BUN contents; increases LG and MG contents; declines ROS and MDA levels; enhances SOD and GSH-Px activities; and regulates the Nrf2 signalling pathway to improve the antioxidant capacity of the body and avoid fatigue.	Exercise-induced fatigue mouse model	5, 10 and 20 mg/kg	[107]
Promotes FasL and IFN-γ expression and significantly promotes NK cell proliferation at 1.6–8.0 μg/mL.	NK cells	8 μg/mL	[108]
Down-regulates mTOR, phosphorylated p70S6 kinase, TLR4, MyD88, NF-κB and p-p65 and shows potential to treat recurrent pregnancy loss.	Rat pregnancy loss model	40 mg/kg	[109]
Down-regulates IL-1β, IL-8 and endothelial adhesion cytokines TF, ICAM1 and VCAM1 in HUVECs.	Human umbilical Vein endothelial cells	50 μM	[110]

Notes: MPO, myeloperoxidase; AMH, adrenal medullary hormone; FSH, follicle-stimulating hormone; SHH, Sonic hedgehog; SMO, smoothened; CYP1B1, cytochrome P450, family 1, subfamily B, polypeptide 1; CYP1A1, cytochrome P450 1A1; UVA, ultraviolet-A; Ki67, nuclear proliferative antigen; PCNA, proliferating cell nuclear antigen; RAW264.7, mouse mononuclear macrophage leukaemia cells; TXNIP, thioredoxin-interacting protein; LA, lactic acid; BUN, blood urea nitrogen; LG, liver glycogen; MG, muscle glycogen; FasL, apoptosis-associated protein factor receptor; IFN-γ, interferon-γ; MyD88, myeloid differentiation factor 88; HUVECs, human umbilical vein endothelial cells.

**Table 12:** Pharmacokinetic Parameters of Hyperoside and Traditional Chinese Medicine Containing Hyperoside.

Detail	C_max_	T_max_	AUC(0-t)	AUC(0-ꝏ)	T_1/2_	MRT	CL	V	Ref.
Hyperoside i.p. 100 mg/kg was administered once.	55,310.9 ng/mL	0.32 h	ND	40535.6 h*ng/mL	ND	0.77 h	2498.5 mL/h/kg	7862.0 mL/kg	[143]
Hyperoside i.g. 100 mg/kg was administered once.	149.0 ng/mL	1.00 h	ND	305.1 h*ng/mL	ND	5.11 h	382,994.1 mL/h/kg	2,290,414.7 mL/kg	
Hyperoside i.v. 2 mg/kg was administered once.	4630.6 ng/mL	0.03 h	ND	748.3 h*ng/mL	ND	0.64 h	2786.7 mL/h/kg	13,835.6 mL/kg	
Hyperoside i.g. 2.5 mL/100 g was administered once.	0.24 ± 0.01 μg/mL	0.39 ± 0.17 h	1344.98 ± 62.31 μg/L*h	1668.05 ± 66.53 μg/L*h	0.04 ± 0.03 h	5.72 ± 0.29 h	ND	ND	[142]
Hyperoside i.g. 2.5 mL/100 g was administered once a day for seven days.	0.32 ± 0.02 μg/mL	2.33 ± 0.52 h	3169.42 ± 678.46 μg/L*h	3831.70 ± 705.26 μg/L*h	0.04 ± 0.01 h	11.87 ± 2.75 h	ND	ND	
*Senecio scandens* water extract i.g. 10 mg/kg was administered once.	1.821 ± 0.179 μg/mL	1 h	0.006 ± 0.003 μg*h/mL	0.023 ± 0.002 μg*h/mL	1.150 ± 0.273 h	ND	ND	ND	[140]
*Cuscuta chinensis* water extract i.g. 2 g/100 g was administered once.	72.03 ng/mL	18 min	ND	5106.86 ng*min/mL	222.58 min	248.83 min	ND	ND	[139]
*Cuscutae Semen* water extract i.g. 15 mg/kg was administered once.	48.44 ± 12.54 μg/L	1.17 ± 0.26 h	159.85 ± 28.71 μg/L*h	177.69 ± 24.88 μg/L*h	6.63 ± 1.41 h	6.41 ± 1.73 h	ND	ND	[144]

Notes: i.p., intraperitoneal injection; i.g., gavage; i.v., tail intravenous injection; ND, not determined.

## Data Availability

Data are available from the authors on request (X.F.).
